# Comparison of the effect of two methods of sucking on pacifier and mother's finger on oral feeding behavior in preterm infants: a randomized clinical trial

**DOI:** 10.1186/s12887-022-03352-9

**Published:** 2022-05-18

**Authors:** Fatemeh Shaki, Parvin Aziznejadroshan, Zahra Akbarian Rad, Mohammad Chehrazi, Afsaneh Arzani

**Affiliations:** 1 Student Research Committee, Babol University of Medical Sciences, Babol, Iran; 2 Non-Communicable Pediatric Disease Research Center, Health Research Institute, School of Nursing and Midwifery, Babol University of Medical Sciences, Babol, Iran; 3 Non-Communicable Pediatric Disease Research Center, Health Research Institute, Babol University of Medical Sciences, Babol, Iran; 4 Department of Biostatistics and Epidemiology, School of Public Health, Babul University of Medical Sciences, Babol, Iran

**Keywords:** Finger Sucking, Infant, Premature, Pacifiers, Intensive Care Unit, Neonatal

## Abstract

**Background:**

Oral feeding problems will cause long-term hospitalization of the infant and increase the cost of hospitalization. This study aimed to compare the effect of two methods of sucking on pacifier and mother's finger on oral feeding behavior in preterm infants.

**Methods:**

This single-blind randomized controlled clinical trial was performed in the neonatal intensive care unit of Babol Rouhani Hospital, Iran. 150 preterm infants with the gestational age of 31 to 33 weeks were selected and were divided into three groups of 50 samples using randomized block method, including non-nutritive sucking on mother’s finger (A), pacifier (B) and control (C). Infants in groups A and B were stimulated with mother’s finger or pacifier three times a day for five minutes before gavage, for ten days exactly. For data collection, demographic characteristics questionnaire and preterm infant breastfeeding behavior scale were used.

**Results:**

The mean score of breastfeeding behavior in preterm infants in the three groups of A,B,C was 12.34 ± 3.37, 11.00 ± 3.55, 10.40 ± 4.29 respectively, which had a significant difference between the three groups (*p* = 0.03). The mean rooting score between three groups of A, B, and C was 1.76 ± 0.47, 1.64 ± 0.48, and 1.40 ± 0.90 (*p* < 0.001) respectively. Also, the mean sucking score in groups of A, B and C was 2.52 ± 0.76, 2.28 ± 0.64 and 2.02 ± 0.74 respectively, which had a significant difference (*p* = 0.003), but other scales had no significant difference between the three groups (*P* > 0.05). The mean time to achieve independent oral feeding between the three groups of A, B, C was 22.12 ± 8.15, 22.54 ± 7.54 and 25.86 ± 7.93 days respectively (*p* = 0.03), and duration of hospitalization was 25.98 ± 6.78, 27.28 ± 6.20, and 29.36 ± 5.97 days (*p* = 0.02), which had a significant difference. But there was no significant difference between the two groups of A and B in terms of rooting, sucking, the total score of breastfeeding behavior and time of achieving independent oral feeding (P > 0.05).

**Conclusion:**

Considering the positive effect of these two methods, especially non-nutritive sucking on mother’s finger, on increasing oral feeding behaviors, it is recommended to implement these low-cost methods for preterm infants admitted to neonatal intensive care unit.

**Trial Registration:**

Trial Registration: IRCT, IRCT20191116045460N1. Registered 11 January 2020- prospective registered.

## Background

The advancement of technology has increased the possibility of surviving preterm infants with a very low gestational age [1]. Most preterm infants are born before the development of the cardiovascular, respiratory, central nervous and muscular systems, which is why a significant number of them have serious problems with oral feeding [2]. Feeding and swallowing problems are the obvious and prevalent medical and behavioral factors that preterm infants and their families face in the neonatal intensive care unit (NICU) [3]. Infant feeding consists of three basic components: sucking, swallowing, and breathing. Unlike full-term infants, most preterm infants cannot be bottle-fed or breastfed immediately after birth. This is due to weak muscle tone, lack of development of mechanisms to control oral movements, and a lack of coordination in sucking, swallowing, and breathing. Also, the cardiorespiratory system, central nervous system, and oral muscles of preterm infants have not developed. Oral feeding problems often affect the infant's ability to achieve independent oral feeding, prolong hospitalization, and may lead to long-term feeding problems [4]. Successful feeding of infants not only plays an important role in their survival but also in improving the infant's communication and speech skills [3, 5]. Since the primary feeding behaviors such as sucking and swallowing are prerequisites for secondary behaviors (e.g.; speech), any disorder in these behaviors will have a direct impact on the future development of the infant's speech communication skills [6]. Breastfeeding is a challenge for neonatal nurses trying to prepare preterm infants for discharge from the hospital [7]. Because oral feeding problems cause prolonged hospitalization of preterm infants in the hospital and are costly, evidence-based interventions are needed to facilitate the development of oral motor skills, improve sucking and feeding behaviors in 30 weeks or younger infants, and reduce the length of hospitalization and costs [8]. Sensory and motor interventions are used to increase the efficiency of oral feeding in preterm infants [9]. Various intervention techniques have been used to facilitate oral feeding in preterm infants, the most common of which are: sensory and motor interventions including cheek and chin support, oral stimulation, tactile-kinesthetic and vestibular stimulation [1, 8]. The beneficial effects of non-nutritive sucking (NNS) and oral stimulation on nutritional efficiency include the coordination of suck-swallow-breathe, the development of the sucking reflex, increased weight gain and reduced time for a transmission from gavage to full oral feeding. Oral stimulation has been reported to lead to weight gain and a decrease in hospitalization and an increase in received milk volume [10]. Younesian et al. noted that due to the combined use of the two methods, it is not clear whether NNS or oral massage is more effective [1, 8]. In studies conducted by Say et al. [11] and Mohammadi PirKashani et al. [12], these two methods have been presented separately and have not been compared with each other. It is not clear which method is better and more suitable for clinical use. This study is important because it will (1) assist healthcare providers in clarifying policy related to implementing treatment for preterm infants in appropriate clinical settings and (2) assist in promoting evidence-based practice in the treatment of preterm infants. If these interventions are found to be effective, they could become a routine and standard part of delivery of care to preterm infants in NICU settings, facilitating earlier discharge and reducing costs of care associated with long hospital stay.

Considering the high importance of oral feeding behaviors and their role in feeding preterm infants and since no study has been conducted to compare these two methods (pacifier and mother's finger) using preterm infant breastfeeding behavior scale (PIBBS), this study aimed to compare the effect of two methods of non-nutritive sucking mother's finger (NSMF) and non-nutritive pacifier sucking (NPS) on oral feeding behaviors in preterm infants.

## Methods

### Study design and setting

This single-blind randomized controlled clinical trial study was done from January 2020 to February 2021 to compare the effect of two methods of sucking on pacifier and mother's finger on oral feeding behaviors in preterm infants in a NICU of Rouhani Hospital. NICU of Rouhani Hospital has level III intensive care affiliated with Babol University of Medical Sciences (Babol, Iran) and high-risk pregnancy referral center.

#### Primary Outcome

Breastfeeding Behavior Scale was measured using the PIBBS [13].

#### Secondary Outcome

The time to achieve independent oral feeding on a daily basis was recorded in the demographic characteristics questionnaire for each infant.

**Fig. 1:**
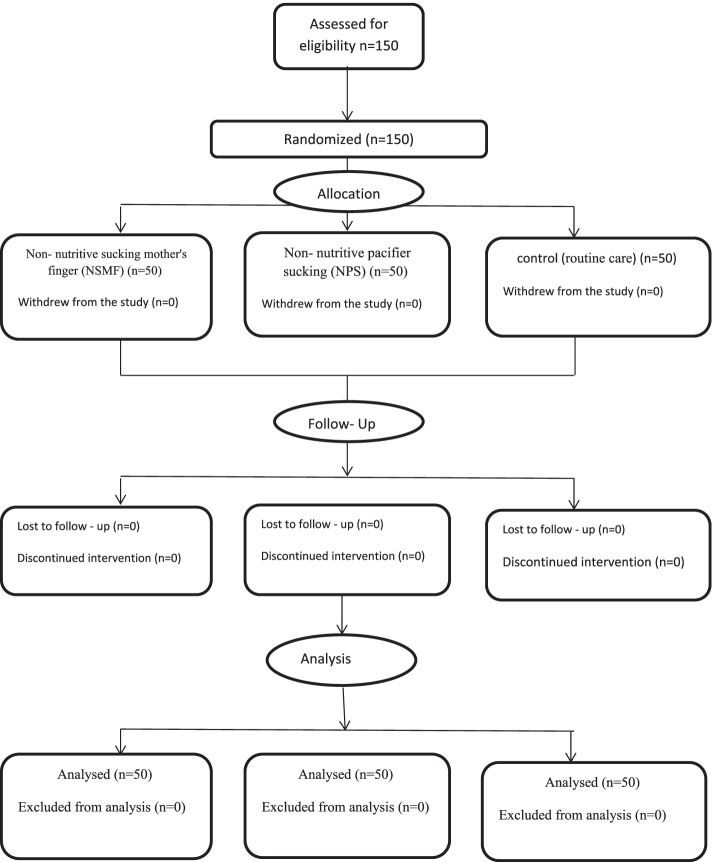
Participant flow diagram according to consolidated standards of reporting trials

This study followed the CONSORT guidelines for reporting RCTs (Fig. 1).

### Sample

150 preterm infants with the gestational age of 31 to 33 weeks and minimum weight of 1350 g fed by gavage were selected and divided into three groups of 50 samples using randomized block method, including NSMF (A), NPS (B), and control (C).

One of the ward nurses (other than the researcher) who was not involved in the patient recruitment process and sample entry was used to hide the random allocation list. First, after reviewing the inclusion criteria and obtaining informed consent, as well as recording the patient's details in a special form, the researcher contacted the nurse who had a random allocation list and the randomization process was performed. Due to the single-blind modality of this study, another nurse (Other than the original researcher), who was trained by the researcher on PIBBS, measured the PIBBS outcome at the end of the study. This nurse was not aware of how infants were allocated to the research groups.

The inclusion criteria included infants with the gestational age between 31 to 33 weeks, minimum weight of 1350 g (appropriate weight to start oral feeding), gavage feeding, no facial and oral anomalies and stabilized clinical conditions.

Ability to suck and to swallow is present by 28 weeks gestation, but infants are not fully coordinated until 32 to 34 weeks [14]. Therefore, preterm infants aged 31–33 weeks were selected who were both close to the time of oral feeding and whose clinical condition was largely stable.

Exclusion criteria included the need to take medication (especially those affecting the baby's central nervous system) and any situation that causes the baby to leave the gavage.

### Sample size

The sample size was calculated based on Mohammadi PirKashani study [12] and considering the measured outcomes using the PIBBS. The intended outcome for calculating the sample size was independent oral feeding.The mean independent oral feeding score in NNS and control groups was 3.8 ± 2 and 5.3 ± 2, respectively [12]. The significance level was considered 0.05 and the power of the test was considered 80%0.50 samples with a drop rate of 15% were required in each group.

$$\mathop n\nolimits_{1} \, = \,\mathop n\nolimits_{2} \, = \,\frac{{(S_{1}^{2} + S_{2}^{2} (Z_{{1 -_{2}^{a} }} + Z_{1 - \beta } )^{2} }}{{(\overline{X}_{1} - \overline{X}_{2} )^{2} }}$$

### Data collection and processing

150 preterm infants with the gestational age of 31 to 33 weeks and minimum weight of 1350 g fed by gavage were selected and divided into three groups of 50 samples using the randomized block method, after applying the inclusion criteria [14]. There was no intervention during the doctors' visit, nursing care or the baby's bedtime. According to the ward physician NSMF and NPS began when the infants reached clinical stability and did not show symptoms such as respiratory arrest and slow heart rate [1]. Kangaroo mother care (KMC) in the ward, routine care was performed. But at the time of the intervention, the infants was in bed and KMC was not taken.

By washing the mother's hands before the intervention, the possibility of germ transmission is reduced. Mothers of group A, after washing their hands with soap and water placed their finger in the baby's mouth three times a day for five minutes before gavage. Two minutes before the intervention, during the intervention and two minutes after the intervention, the infants were examined by a nurse. This procedure was performed three times a day (early morning, noon and afternoon and each time for five minutes) ten days exactly [7, 15].

Mothers of group B, after washing their hands with soap and water gently inserted the pacifier after gently stimulating the baby's lower lip, and continued gentle stimulation of the tongue from the tip to the back until the baby began to suck three times a day before gavage [1]. The pacifiers were “Mina Baby”, the products of Pars Silicon Company made in Iran and had a special cap. Each baby had an exclusive pacifier which was washed with plain water by the mother before each intervention. Mothers were advised to encourage the baby to continue sucking by gently shaking the pacifier if the baby stopped sucking during the intervention. This procedure was performed three times a day (early morning, noon and afternoon and each time for five minutes) ten days exactly [7, 15].

Infants in group (C) received only routine ward care (gavage with breast milk without stimulating the pacifier or the mother's finger). The number and time of interventions performed by mothers were recorded by the first author in the relevant questionnaire [1].

### PIBBS measurement

The primary outcome was the Breastfeeding Behavior Scale that was measured using PIBBS [13] and the secondary outcome was independent oral feeding based on the infant's medical record. PIBBS was used to assess the feeding behavior in preterm infants. The validity and reliability of the PIBBS were reviewed and confirmed in two Nyqvist studies in 1996 and 1999 [13, 16].

PIBBS consists of six parts: Rooting, Areolar grasp, Latched on and fixed to the breast, Sucking, Longest sucking burst, Swallowing, each of which consists of several parts and scores from zero to six. The higher the score, the more complete the feeding behavior. The minimum PIBBS score is zero and the maximum score is 20 [13].

PIBBS were measured in all three groups by a nurse (Other than the original researcher) who was not aware of the allocation of infants to the three groups after 10 days (completion of the intervention). The results were recorded and compared. It was not possible to measure the PIBBS before the intervention because the infant had not yet started oral feeding and the subscales could not be evaluated. A questionnaire was used to collect personal information (age, weight of the infant, time of achieving independent oral feeding, age at the time of discharge, duration of hospitalization, fetal age at birth, Apgar score, sex of the infant).

### Statistical analysis

The statistics advisor performed the data analysis blindly using SPSS Version 18. Descriptive information was shown as frequency, percentage, mean and standard deviation. The PIBBS was reported as Mean ± SD.

Chi-square test was used to examine the relationship between qualitative and One Way ANOVA test was used to compare quantitative variables between the two groups. The Tukey post hoc test was used to compare the outcomes between the three groups after the ANOVA test became significant. The P-value less than 0.05 was considered significant.

### Ethical consideration

The study protocol was approved by the Ethics Committees of Babol University of Medical Sciences (IR.MUBABOL. HRI. REC.1398. 216). The trial is registered in the IRCT20191116045460N1. Before participation in the study, written informed consent was obtained from each child’s primary guardian.

## Results

### Study Subjects

All 150 preterm infants who participated in the study, completed the study (Fig. 1).

The three groups had no significant difference in terms of sex distribution, age at participation time, fetal age, age at onset of oral feeding, birth weight, weight at participation time and Apgar score (*P* > 0.05). However, after the intervention, there was a statistically significant difference between use of either pacifier or finger versus control (*p* = 0.01) and duration of hospitalization (*p* = 0.02) (Table 1).

**Table 1:** Comparison of demographic variables of preterm infants in three groups

Groups^a^			NSMF (A) *n* = 50	NPS (B) *n* = 50	Control (C) *n* = 50	***P* value**
Variables						
Number (%)	Sex	Boy	30(60%)	26(52%)	33(66%)	0.36^b^
		Girl	20(40%)	24(48%)	17(34%)	
	Total		50(100%)	50(100%)	50(100%)	
Mean ± SD	Gestational age (weeks)		31.76 ± 0.71	31.8 ± 0.78	31.62 ± 0.6	0.4^c^
	At birth weight (g)		1644 ± 272.4	1639.7 ± 205.89	1632.4 ± 215.59	0.96^c^
	Age to oral feeding onset (days)		13.8 ± 4.95	12.52 ± 4.52	13.14 ± 5.17	0.42^c^
	Age at enrollment (days)		5.7 ± 5.72	4.9 ± 5.22	4.76 ± 5.82	0.66^c^
	Weight at enrollment (g)		1640 ± 265.3	1615.4 ± 210.75	1618 ± 220.38	0.84^c^
	One-minute Apgar score		7.72 ± 1.53	7.2 ± 0.9	7.52 ± 1.03	0.9^c^
	Five-minute Apgar score		9.15 ± 1.21	9.18 ± 0.85	9.36 ± 0.87	0.61^c^
	Age of Discharge time (days)		18.5 ± 4.81	18.22 ± 4.7	20.92 ± 5.03	0.01^c^
	Duration of hospitalization (days)		25.98 ± 6.78	27.28 ± 6.2	29.36 ± 5.97	0.02^c^

^a^Groups: A. NSMF (non-nutritive sucking mother's finger), B. NPS (non-nutritive pacifier sucking), C. Control

^b^chi2 test

^c^ANOVA test

Tukey test was used to evaluate the variables in pairs between the study groups. In examining the age at the time of discharge, a significant difference was observed between group B and group C (*p* = 0.01) and between group A and group C (*p* = 0.03). however, there was no significant difference between group A and group B (*p* = 0.95).

Also, in terms of the duration of hospitalization, a significant difference was observed between group A and group C (*p* = 0.02). But there was no significant difference between group B and group C (*p* = 0.23) and also between group A and group B (*p* = 0.56) (Table 2).

**Table 2:** Double comparisons of mean and standard deviation age at the time of discharge and duration of hospitalization in preterm infants in the study groups

Groups^a^	NSMF (A) (Mean ± SD) *n* = 50	NPS (B) (Mean ± SD) *n* = 50	Control (C) (Mean ± SD) *n* = 50	*P* value^b^
Variables				
Age of discharge time(day)	-	18.22 ± 4.70	20.92 ± 5.03	0.01
	18.50 ± 4.81	-	20.92 ± 5.03	0.03
	18.50 ± 4.81	18.22 ± 4.70	-	0.95
Duration of hospitalization (day)	-	27.28 ± 6.20	29.36 ± 5.97	0.23
	25.98 ± 6.78	-	29.36 ± 5.97	0.02
	25.98 ± 6.78	27.28 ± 6.20	-	0.56

^a^Groups: A. NSMF (non-nutritive sucking mother's finger), B. NPS (non-nutritive pacifier sucking), C. Control

^b^Tukey test

The mean rooting score in group A was higher than the two groups B and C and this difference was significant (*P* < 0.05).

The mean score of sucking and PIBBS score were significantly different between the three groups (*P* < 0.05), and it was higher in group A than group B, and also in group B than group C.

The time to achieve independent oral feeding was significantly different between the three groups (*P* < 0.05). The shortest time was o.bserved in group A, group B and group C, respectively.

Other scales of the questionnaire (Areolar grasp, Latched on and fixed to the breast, Longest sucking burst and Swallowing) had no significant difference between the three groups (p > 0.05) (Table 3).

**Table 3:** Comparison of mean and standard deviation PIBBS score in the three groups

Groups^a^	NSMF(A) *n* = 50	NPS (B) *n* = 50	Control (C( *n* = 50	*P* value^b^
Variables				
Rooting	1.76 ± 0.47	1.64 ± 0.48	1.30 ± 0.67	< 0.001
Areolar grasp	2.38 ± 0.85	2.20 ± 0.92	1.96 ± 1.06	0.09
Latched on and fixed to the breast	1.66 ± 0.68	1.46 ± 0.76	1.40 ± 0.90	0.23
Sucking	2.52 ± 0.76	2.28 ± 0.64	2.02 ± 0.74	0.003
Longest sucking burst	2.32 ± 0.84	1.96 ± 0.96	2.10 ± 1.21	0.21
Swallowing	1.70 ± 0.46	1.52 ± 0.61	1.60 ± 0.49	0.23
Total score of PIBBS	12.34 ± 3.37	11.00 ± 3.55	10.40 ± 4.29	0.03
Time to achieve independent oral nutrition (day)	22.12 ± 8.15	22.54 ± 7.54	25.86 ± 7.93	0.03

^a^Groups: A. NSMF (non-nutritive sucking mother's finger), B. NPS (non-nutritive pacifier sucking), C. Control

^b^ANOVA test

The result of the Tukey test in examining the rooting, sucking, the total score of breastfeeding behavior and the time of achieving independent oral feeding, showed a significant difference was observed between group A and group C and also between group B and group C (*P* < 0.05), but no significant difference was found between group A and group B (*p* > 0.05) (Table 4).

**Table 4:** Double comparisons of the mean and standard deviation of the PIBBS scores

Groups^a^	NSMF (A) *n* = 50	NPS (B) *n* = 50	Control (C) *n* = 50	*P* value
Variables				
Rooting	1.80 ± 0.40	_	1.32 ± 0.68	< 0.001
	_	1.64 ± 0.48	1.32 ± 0.68	0.007
	1.80 ± 0.40	1.64 ± 0.8	_	0.52
Sucking	2.52 ± 0.75	_	2.05 ± 0.77	0.002
	_	2.30 ± 0.65	2.05 ± 0.77	0.16
	2.52 ± 0.75	2.30 ± 0.65	_	0.21
Total score of PIBBS	12.34 ± 3.37	_	10.40 ± 4.29	0.02
	_	11.00 ± 3.55	10.40 ± 4.29	0.70
	12.34 ± 3.37	11.00 ± 3.55	_	0.75
Time to Achieve Independent Oral Nutration (Days)	22.12 ± 8.15	_	25.86 ± 7.93	0.04
	_	22.54 ± 7.54	25.86 ± 7.93	0.09
	22.12 ± 8.15	22.54 ± 7.54	_	0.96

^a^Groups: A. NSMF (non-nutritive sucking mother's finger), B. NPS (non-nutritive pacifier sucking), C. Control

**Fig. 2:**
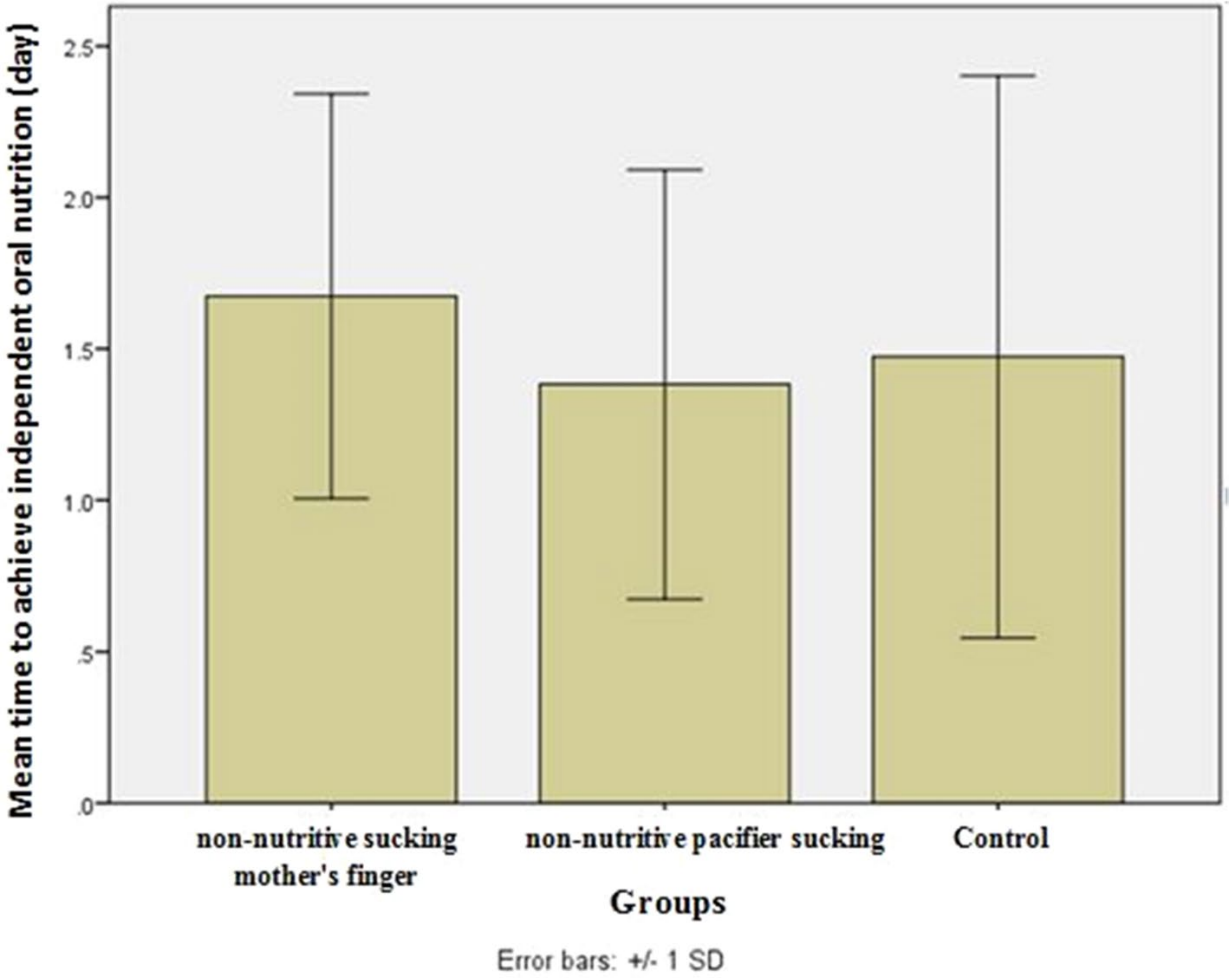
Comparison of the effect of three methods (non-nutritive sucking mother's finger (NSMF), non-nutritive pacifier sucking (NPS) and control group) on the time of achieving independent oral feeding in preterm infants

According to the chart above, the infants achieved independent oral feeding in a shorter period by NSMF and NPS than the control group (Fig. 2).

## Discussion

We hypothesized that non- nutritive sucking mother's finger or non- nutritive pacifier sucking versus no intervention accelerate the attainment of independent oral feeding through a faster maturation of infants’ oral feeding skills (OFS). The results of the present study showed that the mean PIBBS score, mean rooting and sucking score, time of independent oral feeding and duration of hospitalization in preterm infants were significantly different between the three groups. The highest mean PIBBS score was observed in the NSMF, followed by the NPS compared to the control group, respectively. However, there was no significant difference in terms of mean rooting, sucking, PIBBS score and time of achieving independent oral feeding between the two groups of NSMF and NPS. In other words, NSMF and NPS compared to the control group can improve the infant's oral feeding behaviors and also reduce the time to achieve independent oral feeding and shorten the duration of hospitalization in preterm infants.

In a study conducted by Say et al. in 2018 on the effect of pacifier on the transition time from gavage to breastfeeding in preterm infants, they found that the transition time to full breastfeeding was significantly shorter in the pacifier group compared to the control group. They concluded that giving pacifiers to preterm infants during gavage feeding can reduce the duration of transition to oral feeding and the duration of hospitalization [11]. These results are consistent with the findings of the present study.

In 2013, Keshavarz et al. examined the effect of non-nutritive sucking on the weight gain of preterm infants admitted to NICU. Their results showed that due to the positive effect of pacifier sucking on the weight gain of preterm infants, it is recommended to use this method during the gavage of preterm infants at NICU [17]. The results of this study are similar to the present study.

In an RCT in 2018, Mohammadi Pirkashani et al. examined the effect of NSMF to tolerate feeding and achieve independent oral feeding in preterm infants. They reported that NSMF could be effective in improving feeding tolerance and accelerating the achievement of independent oral feeding in preterm infants, resulting in early hospital discharge [12]. These results are consistent with the findings of the present study in terms of time to achieve independent oral feeding. Besides, in our study, there was a significant difference between the mean score of PIBBS in the NSMF and NPS groups in preterm infants. It means NSMF compared to NPS group can cause an increase in oral feeding behaviors in preterm infants, which was not mentioned in other studies.

In 2019, Mahmoodi et al. examined the effect of premature infant oral motor intervention (PIOMI) on the early onset of oral feeding and the feeding improvement in preterm infants. Their results showed that the intervention group achieved independent feeding earlier than the control group. In addition, the duration of hospitalization in the intervention group was shorter than in the control group [18]. Despite the differences in the type of interventions, the results of the two studies are consistent.

In 2013 Vashani et al. studied the effect of non-nutritive sucking on weight gain and duration of hospitalization in preterm infants. They found that non-nutritive sucking can significantly reduce the duration of hospitalization [19]. The result of this study is consistent with the present study.

In a study conducted by Valizadeh et al. in 2014, they compared the effect of two techniques of non-nutritive sucking and oral massage on the time of achieving independent oral feeding and the duration of hospitalization in preterm infants admitted to NICU. Their results indicated that the two intervention groups achieved independent oral feeding significantly earlier than the control group. But there was no statistically significant difference between the time of achieving independent oral feeding in the two groups of oral massage and non-nutritive sucking. They concluded that the two techniques of non-nutritive sucking and oral massage can shorten the time to achieve independent oral feeding in preterm infants, but these two methods have no superiority over each other [1]. The results of this study, except for the oral massage intervention that was not used in our study, are consistent with the present study for non-nutritive sucking and its effect on independent oral feeding.

Using the mother finger as a non-nutritive sucking based on newborn individualized developmental care and assessment program (NIDCAP) engaging parents in infant care, is a practical way to increase independent oral feeding skills in premature infants. On the other hand, the mother will use her finger to convey the motherly feeling and comfort to the infant [1].

A study by Lau et al. in 2012, on performing interventions to improve the oral feeding of preterm infants, showed that swallowing is an effective intervention in facilitating the process of achieving independent oral feeding, but sucking is not. The researchers believed that the benefit of swallowing can be due to the preterm maturation of infants' oral feeding skills [20]. The results of the Lau et al. study are not consistent with the present study. Because in our study, sucking skills in non-nutritive sucking groups were better than the control group and swallowing skills had no difference. Probably the reason for this discrepancy could be the difference in sample size and the different research tools used to examine the Breastfeeding Behavior Scale, the age of the preterm infants and their weight. In the Lau study, 70 infants with a very low birth weight of 540-1290 g and gestational age of 24–33 weeks were studied, while the present study examined 150 preterm infants with the gestational age of 31 to 33 weeks and minimum weight of 1350 g.

In their study in 2019, John et al. compared non-nutritive breastfeeding in the intervention group with a non-nutritive finger sucking in the control group during gavage feeding as facilitators of oral feeding skills in preterm infants. The results suggested that the infants in the intervention group showed the faster transition of non-nutritive sucking maturity stages and had more sucks per burst during breastfeeding than the control group [21]. The findings of the above study are not consistent with the results of the present study. In our study, there was a significant difference between the mean scores of PIBBS in the non-nutritive finger sucking group, the pacifier group and the control group in preterm infants. It means non-nutritive sucking of the mother’s finger group had better oral feeding behaviors compared to the pacifier and control groups. While in John's study, non-nutritive breastfeeding accelerated oral feeding skills in preterm infants rather than non-nutritive finger sucking [21]. This inconsistency can be due to using different interventions. However, there is a consistency between the two studies in terms of the number of sucks per burst, meaning that both non-nutritive sucking of the mother's finger and non-nutritive sucking of the mother's breast resulted in the increased number of sucks per burst compared to the control group.

We had no particular barriers to implementing or using this method. No adverse effects such as sepsis, oral infection, oral traumas, Loss of oxygenation, apnoea or bradycardia episodes that require intervention from the caregiver (stimulation, oronasal suction, assisted ventilation) or death during initial hospital stay were reported. Many studies did report adverse effects of apnoea and bradycardia that were self-resolving and did not require intervention other than cessation of the oral stimulation intervention.

To promote or reinforce NSMF technique, only the infant mother should be encouraged to participate in care. This technique can be easily used in centers where NIDCAP is run.

## Conclusion

According to the results of the present study, there was a significant difference between the three groups in the mean PIBBS score, mean rooting and sucking score, time to achieve independent oral feeding and duration of hospitalization in preterm infants. The highest mean PIBBS score belonged to NSMF group, then the NPS had the highest mean score compared to the control group. There was no significant difference between NSMF group and NPS group in terms of mean rooting score, sucking score, PIBBS score and time of achieving independent oral feeding. In other words, NPS and NSMF can improve the infant's oral feeding behaviors and reduce the time to achieve independent oral feeding and shorten the duration of hospitalization in preterm infants. As a result, the positive effect of these two non-nutritive sucking methods can be useful in reducing the mortality rate of preterm infants. In addition, parental involvement in taking care of preterm infants and creating and maintaining an emotional relationships between parents and infants, along with low-cost and low-risk non-nutritive finger sucking interventions are recommended to improve oral feeding behaviors in preterm infants. This technique (NSMF) can be easily used in centers where NIDCAP is run. The role of the father can also be used in future studies. This useful study will add value to methods of providing non-nutritive suck able in limited environments as well as in developed economies.

### Study Limitations

Since this research was conducted in an educational and medical center, there was an age limitation (infants with the gestational age of 31–33 weeks), which can lead to different research results.

## Data Availability

The datasets generated and analyzed during the current study are not publicly available due to an agreement with the participants on the confidentiality of the data but are available from the corresponding author on reasonable request.

## Abbreviations

PIBBS: Preterm Infant Breastfeeding Behavior Scale
NICU: Neonatal Intensive Care Unit
NPS: Nonnutritive Pacifier Sucking
NSMF: Nonnutritive Sucking Mother's Finger
NNS: Non-Nutritive Sucking
SD: Standard Deviation
MD: Mean Difference
ANOVA: Analysis of Variance
chi2: Chi-square
CONSORT: Consolidated Standards of Reporting Trials
RCT: Randomized Controlled Trial
APGAR: Appearance, Pulse, Grimace, Activity, Respiration
PIOMI: Premature Infant Oral Motor Intervention
NIDCAP: Newborn Individualized Developmental Care and Assessment Program
KMC: Kangaroo Mother Care
OFS: Oral Feeding Skills
